# Adaptive Governance: Empowering Data Sharing for Public Health Impact - Insights from North Carolina Department of Health and Human Services

**DOI:** 10.23889/ijpds.v9i5.2570

**Published:** 2024-09-18

**Authors:** Daniel P. Riggins, Jill Inderstrodt, John Price, Shaun J. Grannis, Titus Schleyer, Jennifer Crago, Stephen O'Brien, Brian E. Dixon

**Affiliations:** 1 Regenstrief Institute, Center for Biomedical Informatics; 2 Indiana University Richard M. Fairbanks School of Public Health; 3 Indiana University School of Medicine

Indiana faces significant challenges in maternal and infant health, ranking 45th worst in infant mortality with a rate of 6.5 deaths per 1,000 live-births, and 21st in stillbirths. Indiana further has the 3rd highest rate of maternal mortality with 44 deaths per 100,000 live-births. Despite these alarming statistics, comprehensive surveillance of maternal and infant health is absent, and data often remains fragmented across disparate information systems.

In response, the Indiana University Fairbanks School of Public Health and Regenstrief Institute secured a cooperative agreement through the CDC's Pregnant People-Infant Linked Longitudinal Surveillance (PILLARS) program, collaborating with the Indiana Health Information Exchange, Indiana Department of Health, and Marion County Public Health Department. The initiative aims to enhance Indiana's infrastructure for surveillance of maternal and child health (MCH) using electronic health records (EHRs), public health, and administrative data. Key efforts have included development, validation, and application of linkage algorithms across records for mothers and children; integration of case data across data sources; design of routine surveillance reports; and design of longitudinal studies to examine determinants and outcomes in MCH populations. Using deterministic linkage algorithms, we have created over 800,000 mother-infant dyads; this number will expand with the implementation of probabilistic approaches. We also have developed a computational phenotype for identifying cases of pregnancy in the EHR even when not explicitly flagged in the EHR. Future efforts will build on this infrastructure to draw from additional public health data sources and/or expand surveillance efforts to include prioritized MCH outcomes.

